# PSMA Targeted Molecular Imaging and Radioligand Therapy for Prostate Cancer: Optimal Patient and Treatment Issues

**DOI:** 10.3390/curroncol30080529

**Published:** 2023-08-01

**Authors:** Seiji Hoshi, Kei Yaginuma, Satoru Meguro, Akifumi Onagi, Kanako Matsuoka, Junya Hata, Yuichi Sato, Hidenori Akaihata, Masao Kataoka, Soichiro Ogawa, Motohide Uemura, Yoshiyuki Kojima

**Affiliations:** Departments of Urology, School of Medicine, Fukushima Medical University, 1 Hikarigaoka, Fukushima 960-1295, Japan; uro-yagi@fmu.ac.jp (K.Y.); s-meguro@fmu.ac.jp (S.M.); onagi@fmu.ac.jp (A.O.); kanaco@fmu.ac.jp (K.M.); akju826@fmu.ac.jp (J.H.); ysato@fmu.ac.jp (Y.S.); hakai@fmu.ac.jp (H.A.); masaoka@fmu.ac.jp (M.K.); soh@fmu.ac.jp (S.O.); muemura@fmu.ac.jp (M.U.); ykojima@fmu.ac.jp (Y.K.)

**Keywords:** theranostics, PSMA, prostate cancer, molecular imaging, radioligand therapy

## Abstract

Theranostics (therapy + diagnosis) targeting prostate-specific membrane antigen (PSMA) is an emerging therapeutic modality that could alter treatment strategies for prostate cancer. Although PSMA-targeted radioligand therapy (PSMA-RLT) has a highly therapeutic effect on PSMA-positive tumor tissue, the efficacy of PSMA-RLT depends on PSMA expression. Moreover, predictors of treatment response other than PSMA expression are under investigation. Therefore, the optimal patient population for PSMA-RLT remains unclear. This review provides an overview of the current status of theranostics for prostate cancer, focusing on PSMA ligands. In addition, we summarize various findings regarding the efficacy and problems of PSMA-RLT and discuss the optimal patient for PSMA-RLT.

## 1. Introduction

Prostate cancer is the second most commonly diagnosed malignant disease and the fifth leading cause of death among men in the world [1]. Localized prostate cancer has a good prognosis with treatment such as surgery and radiation, but recurrent or metastatic cancer leads to a lethal disease called castration-resistant prostate cancer (CRPC). Drugs, such as second-generation antiandrogens (enzalutamide [2], abiraterone [3], apalutamide [4], darolutamide [5]), taxanes (docetaxel [6], cabazitaxel [7]), PARP inhibitors (olaparib [8]), and radium-223 [9], have prolonged prognosis in CRPC patients, but CRPC remains difficult to cure. However, the situation is changing with the recent discovery of a cancer-specific protein called prostate-specific membrane antigen (PSMA). There has been remarkable development in “theranostics”, in which antibodies or small molecular compounds that bind to PSMA are coupled with the diagnostic emitter or therapeutic alpha- or beta-emitters to diagnose or treat prostate cancer, respectively. Then, in March 2022, the US Food and Drug Administration (FDA) approved ^177^Lu-PSMA-617, a radioligand therapy for PSMA (PSMA-RLT), based on the results of the VISION trial [10]. In the VISION trial, ^177^Lu-PSMA-617 extended prognosis in heavily pretreated CRPC patients. However, although ^177^Lu-PSMA-617 offers a longer-term prognosis for some patients, the overall survival difference was only 4 months compared to the control group, and hence CRPC remains a fatal disease. In addition, despite the inclusion of only PSMA-positron emission tomography (PET) positive patients in this study, some patients do not show an adequate treatment response. Therefore, ^177^Lu-PSMA-617 does not always benefit all CRPC patients, and it is unclear which patients would benefit from this therapy. This review provides an overview of the current status of diagnostic and therapeutic agents and milestone studies in the rapidly developing PSMA-RLT. We also review the therapeutic effects and side effects of PSMA-RLT and outline the currently developing PSMA therapies, including combination therapy and alpha-emitter therapy. Finally, the optimal patient population for PSMA-RLT is discussed based on the current evidence.

## 2. PSMA-PET Imaging in Prostate Cancer

Several tracers are used in PSMA-PET. The most commonly reported PSMA-PET diagnostic agent is ^68^Ga-PSMA-11. Other ligands and 18F-labeled tracers are also outlined here. The current status of PSMA-PET images in radiological reading is also discussed.

### 2.1. PSMA-Targeted Monoclonal Antibody

Horoszewicz et al. developed 7E11, a mouse-derived monoclonal antibody (mAb) that specifically binds to PSMA-positive cells, as the first PSMA-binding antibody for nuclear medicine [11]. However, PET drugs with 7E11 antibodies were not suitable for RLT due to their long persistence in the body and poor migration to bone and microtissues [12]. To improve these problems, the J591 antibody was developed, which showed faster clearance than 7E11 and was considered to be useful because of its high specificity for the target [11]. However, Phase I/II imaging studies using 89Zr-labeled J591 concluded that the mAb has practical limitations in terms of clearance [13]. Drug development for short-lived antibodies derived from single-chain fragments is still underway.

### 2.2. Low Molecular Weight (LMW) PSMA Agents

Low molecular weight (LMW) PSMA ligands are typically based on a skeleton containing a specific PSMA-binding entity (urea-based), a linker, and a chelator for labeling with a radionuclide. This LMW compound is combined with a radionuclide for PET and used as a PSMA-PET agent. The European Association of Urology (EAU) [14,15] and National Comprehensive Cancer Network (NCCN) [16] guidelines recommend the use of PSMA-PET for lesion evaluation in biochemical recurrence patients, staging of high-risk localized prostate cancer, and expression evaluation in metastatic prostate cancer before PSMA-RLT. Herein, three widely used PSMA-PET agents are described.

#### 2.2.1. ^68^Ga-PSMA-11

^68^Ga-PSMA-11 is a drug approved by the FDA on 1 December 2020. The most widely used PSMA-PET agent is created by synthesizing PSMA-11 with [68]-gallium extracted from a germanium/gallium (Ge/Ga) generator. It was approved by the European Medicines Agency (EMA) on 2 February 2023 as gozetotide (Locametz^TM^). It is approved for diagnosis of recurrence after radical treatment, staging of localized prostate cancer, and metastatic prostate cancer. ^68^Ga-PSMA-11 shows high accumulation in prostate cancer, but it should be discontinued in the diagnosis of perineural recurrence due to urinary excretion [17]. This point is reported to be reduced by the administration of furosemide, hydration, and urination [18,19,20]. If a Ge/Ga generator is available, it is relatively easy to create, but the short half-life of ^68^Ga [21] makes it unsuitable for delivery.

#### 2.2.2. ^18^F-PSMA-1007

In general, agents using 18F have a longer half-life than ^68^Ga agents, making them suitable for image evaluation at later time points and mass production. ^18^F is generally synthesized in cyclotrons and has a longer half-life than ^68^Ga [21], which may be superior in production and supply, but production facilities are limited due to the need for huge cyclotrons. ^18^F-PSMA-1007 is characterized by biliary excretion and low urinary excretion, which is considered an advantage in the evaluation of local lesions [22]. However, it is highly concentrated in the liver, which may mask liver metastatic lesions, and may not be suitable for patients with severe hepatic dysfunction [23]. Some reports suggest that it is advantageous in the diagnosis of recurrence after total prostatectomy because of its lower urinary excretion compared to other agents, but others report that there was no difference in diagnostic performance [22]. Although this drug has not been approved by the FDA and EMA, it is currently being compared to Ga-PSMA-11 [24] and is a promising agent for future approval.

#### 2.2.3. ^18^F-DCFPyL

^18^F-DCFPyL is an agent approved by the FDA in 1/DEC/2020 as piflufolastat^TM^; it is not yet approved by the EMA. The indications are the same as for ^68^Ga-PSMA-11, which is approved for the diagnosis of recurrence after radical therapy, staging of localized prostate cancer, and metastatic prostate cancer. It is excreted from the urine and liver, but its accumulation in the liver is reported to be milder than that of ^18^F-PSMA-1007, and it may be safer than ^18^F-PSMA-1007 in patients with hepatic dysfunction [23].

### 2.3. Reading and Diagnosis of PSMA-PET

#### 2.3.1. Diagnostic Ability of PSMA-PET

Most of the current reports of PSMA-PET were performed using PSMA-PET/CT. PSMA-PET has a lot of evidence for primary staging evaluation in localized intermediate- and high-risk prostate cancer and diagnosis of biochemical recurrence (BCR) after radical prostatectomy (RP) or radiotherapy, but there are many challenges for diagnosis in metastatic prostate cancer and CRPC. In a prospective phase III trial of 764 intermediate- and high-risk prostate cancer patients with RP, the diagnostic sensitivity and specificity of ^68^Ga-PSMA-11 PET/CT for lymph node metastatic stage were 0.40 (95% CI: 0.34–0.46) and 0.95 (95% CI: 0.92–0.97), respectively [25]. In another meta-analysis of 37 studies on the primary staging by ^68^Ga-PSMA-11 PET/CT, the sensitivity and specificity for the diagnosis of lymph node metastases were 77% and 97%, respectively, in a patient-based analysis [26]. PSMA-PET for local staging in the primary staging of prostate cancer is not approved and is still experimental. PSMA-PET/MRI has been reported to be more effective than PET/CT for primary staging [27] and has been noted to have better diagnostic ability for T3a and T3b [28]. If PSMA-PET/MRI enables TNM classification staging in a single scan, it should be potentially beneficial to patients, and future studies are warranted. We are currently evaluating a prospective comparative study of multi-parametric MRI and PSMA-PET/MRI for preoperative evaluation of local staging for localized prostate cancer (jRCTs022220021), and the results will be reported in the future. An attempt to fusion-biopsy using PSMA-PET/MRI for men with elevated PSA (NCT03187990) is ongoing, and future results are expected. With regard to the primary staging of bone metastases, 12 studies, including a systematic review, reported that the sensitivity and specificity of PSMA-PET/CT for the primary staging of high-risk localized prostate cancer were better than CT/bone scintigraphy on both sensitivity (median sensitivity per lesion 33–92% and per patient 66–91%) and specificity (median specificity per lesion 82–100% and per patient 67–99%) [29]. In a prospective comparative trial (ProPSMA) of 302 patients with high-risk localized prostate cancer prior to RP or radiation therapy, the diagnostic accuracy for lymph node or distant metastasis of ^68^Ga-PSMA-11 PET/CT was 27% (95% CI: 23–31) higher than CT and bone scan (92% (95% CI: 88–95) vs. 65% (95% CI: 88–95)) [30]. Thus, in localized prostate cancer, Ga-PSMA-PET has a high metastasis detection rate, but it remains unclear whether reclassification of the clinical stage based on these imaging results alters the patient’s post-treatment prognosis. In persistently elevated PSA after RP patients, the detection rate of metastasis of PSMA PET/CT was 42, 58, 76, and 95% in patients with PSA levels of 0–0.2, 0.2–1, 1–2, and >2 ng/mL, respectively [26]. In a prospective multicenter study of 323 patients with BCR, PSMA PET/CT significantly reduced the number of patients with unknown recurrence sites (77 vs. 19%, *p* < 0.001) and significantly increased the number of patients with metastatic disease (11 vs. 57%) compared to conventional imaging (CT and bone scan), and as a result, the treatment plan was changed in 62% of patients [31]. PSMA-PET was indicated to have a potentially important role in treatment planning for salvage radiation therapy after curative treatment [32], but further reports are needed. The optimal use of PSMA-PET as a pre-treatment patient selection tool for metastatic prostate cancer prior to PSMA-RLT remains unclear. In the TheraP phase II trial [33] of ^177^Lu-PSMA-617 treatment for CRPC patients, 29 of 291 patients (10%) were excluded from the trial due to low ^68^Ga-PSMA-11 accumulation. Due to tumor heterogeneity in metastatic prostate cancer, PSMA-PET accumulation is expected to differ between patients and tumors. There are no established criteria for deciding which metastatic lesions are eligible for PSMA-RLT, and therefore, it is necessary to explore the optimal selection criteria based on actual clinical studies in the future.

#### 2.3.2. Development of Standardized Image Interpretation

As PSMA-PET has become more widely used, discrepancies in findings between radiologists have increased due to uptake in benign lesions and non-prostatic malignancies [34,35,36,37,38,39]. Three guidelines (criteria) are currently reported to resolve this problem: PROMISE criteria [40], PSMA-RADS [41], and EANM criteria [17]. The external validity of these criteria has recently been evaluated, and although they have good reproducibility in the assessment of ^68^Ga-PSMA-11, there are factors that cause disagreement among readers, and further research is needed to standardize the reading of PSMA-PET imaging [42]. Based on these studies, the EANM standardized reporting guidelines v1.0 for PSMA-PET (E-PSMA guideline) were suggested [23]. The guideline recommends that PSMA accumulation be described on a four-point scale (Table 1) and that TNM stages be described by molecular imaging TNM (miTNM) classification according to the PROMISE criteria (Table 1). The guidelines also indicate that PSMA-PET can be positive in other malignancies such as renal, lung, breast, and liver cancers, as well as in ganglia, benign bone lesions, benign neurogenic tumors, and sarcoidosis. Despite these efforts to standardize reading, it is important to understand that these guidelines are primarily intended for biochemical recurrence and not for metastatic prostate cancer.

#### 2.3.3. PSMA-PET Imaging in Metastatic Prostate Cancer

The role of PSMA-PET imaging in the treatment of progressive prostate cancer is under development. In non-metastatic CRPC (nmCRPC) with conventional imaging modalities, PSMA-PET has been proven to detect metastatic sites [43], but it is unclear whether this subgroup can help identify who would benefit from the stratification of patients with this imaging PSMA-PET is also currently being investigated for use in the evaluation of treatment of metastatic prostate cancer, and the PSMA PET progression (PPP) criteria have been suggested [44]. This set of criteria defines treatment responses in three distinct phases: (1) the appearance of two or more new PSMA-positive distant lesions; (2) the appearance of one new PSMA-positive lesion plus consistent clinical or laboratory data and recommended confirmation by biopsy or correlative imaging within 3 months of PSMA PET; and (3) an increase of ≥30% in size or uptake plus consistent clinical or laboratory data and confirmation by biopsy or correlative imaging within 3 months of PSMA PET. This criteria set should be considered when reporting PSMA-PET in patients receiving systemic therapy, but its efficacy needs to be validated in the future. PSMA-PET is also used for pre-treatment evaluation of RLT for advanced prostate cancer, but there are no clear criteria at present. Two prospective comparative studies on PSMA-RLT have been completed at this time: the VISION trial [10] and the TheraP trial [33]. Both trials used PSMA-PET/CT to identify patients with high PSMA expression, but the thresholds used to define indications differed among the trials, and no fixed criteria have been established; thus, future studies are needed.

## 3. PSMA-Radioligand Therapy (PSMA-RLT)

The increasing use of PSMA-PET imaging for diagnosis has shown that it is possible to identify PSMA-PET tracer uptake in many prostate cancer metastatic lesions. Therefore, there have been attempts to replace a radioisotope (RI) for diagnosis with a therapeutic RI that emits α- or β-rays (theranostics; therapy + diagnosis). Although α- and β-rays provide high energy to the tumor, their range is short [45], and a large number of radionuclides must be taken up in the target tissue to achieve a therapeutic effect. For this reason, LMW PSMA ligands with excellent tissue translocation [46,47] are often used for RLT. This is also a factor in the use of LMW PSMA ligands since poor clearance of PSMA ligands and prolonged residence of RI in the blood can lead to the appearance of strong hematologic toxicity. Comparing α- and β-rays, α-rays have higher energy than β-rays and can cause efficient DNA double-strand breaks (DSB). However, the disadvantage of α-rays is that they can only destroy accumulated cells and, thus, cannot be expected to have the crossfire effect that β-rays have, which can damage PSMA-negative cells adjacent to PSMA-positive cells [45]. The typical α- and β-ray emitting nuclides, including those in the basic research stage, are listed in Table (Table 2). The current PSMA-RLT process is to identify therapeutic targets in CRPC patients with diagnostic tracers (^68^Ga-PSMA-11 or ^18^F-DCFPyL) and to treat metastatic sites with PSMA ligands (PSMA-617, PSMA I&T, etc.) conjugated with β ([177]-lutetium, ^177^Lu) or α ([225]-actinium, ^225^Ac) emitter. Here, we summarize the current efficacy, effectiveness, and problems with PSMA-RLT, focusing on ^177^Lu-PSMA-617, which was recently approved by the FDA and had the best therapeutic results, and introduce the currently developed therapies.

### 3.1. ^177^Lu-PSMA-617

#### 3.1.1. Summary Clinical Trials of ^177^Lu-PSMA-617

The radiopharmaceutical for PSMA-RLT that is supported by the most solid data is 177Lu-PSMA-617. This agent was first started in patients in 2014 [48] and has shown promising results in CRPC patients with multiple treatment histories [49]. There are two milestone studies on this agent.

The TheraP trial, a randomized phase II trial, was first conducted by the Hofman, M. S. et al. group in Australia [33]. Since cabazitaxel was considered the next appropriate standard of care (SoC) after docetaxel in this trial, CRPC patients highly selected by ^68^Ga-PSMA-11 and ^18^F-FDG PET/CT scans were randomized to receive ^177^Lu-PSMA-617 (6 GBq intravenous injection every 6 weeks for up to 6 cycles) or cabazitaxel (20 mg/m^2^ for up to 10 cycles). The primary endpoint was prostate-specific antigen (PSA) response defined by a reduction of at least 50% from baseline. Primary endpoints have been achieved (^177^Lu-PSMA-617 group vs. cabazitaxel group, PSA responses; 66 vs. 37% by intention to treat; difference 29% (95% CI: 16–42; *p* < 0·0001; and 66 vs. 44% by treatment received; difference 23% [9,10,11,12,13,14,15,16,17,18,19,20,21,22,23,24,25,26,27,28,29,30,31,32,33,34,35,36,37]; *p* = 0·001)). However, the study results require careful interpretation because the primary endpoint of the study was at least a 50% reduction in PSA; in addition, the dose of 20 mg/m^2^ cabazitaxel was lower than the 25 mg/m^2^ in the TROPIC [7] and CARD trials [50].

An open-label phase III trial (VISION trial) was subsequently conducted by Sartor, O. et al. in the United States. This study compared ^177^Lu-PSMA-617 radioligand therapy with SoC in metastatic CRPC (mCRPC) patients with metastases expressing PSMA on PET/CT and previously treated with at least one androgen receptor axis-targeted therapy agent (ARAT) and one or two taxanes. Imaging-based progression-free survival (PFS) and overall survival (OS) were alternative primary endpoints. Eligible patients had at least one PSMA-positive metastatic lesion that exceeded liver parenchymal uptake on ^68^Ga-PSMA-11 PET/CT, a lymph node with a short axis greater than 2.5 cm, a metastatic solid organ lesion with a short axis greater than 1.0 cm, and a metastatic bone lesion with a soft tissue component greater than 1.0 cm in the short axis. More than 800 patients were randomized. ^177^Lu-PSMA-617 significantly prolonged both imaging-based PFS by 5.3 months (median, 8.7 vs. 3.4 months) and OS by 4 months (median, 15.3 vs. 11.3 months) compared to SOC. Although more Grade 3 or higher adverse effects (AEs) occurred with the ^177^Lu-PSMA-617 group than the SoC group (52.7 vs. 38.0%), there was no significant effect on the quality of life. ^177^Lu-PSMA-617 was shown to be a valuable treatment option in this mCRPC population. These results led to FDA and EMA approval of ^177^Lu-PSMA-617. Clinical trials are currently underway for ^177^Lu-PSMA-617, including an open-label randomized controlled trial for patients with metastatic hormone-sensitive prostate cancer (NCT04720157) and a phase II trial for castration-sensitive prostate cancer with oligo metastases (NCT04443062, NCT05079698), and the indication is expected to expand.

#### 3.1.2. Predictors of Treatment Effectiveness of ^177^Lu-PSMA-617 and Limitations

In the VISION study, ^177^Lu-PSMA-617 prolonged the prognosis of heavily pretreated CRPC patients, but the prolonged duration was only 4 months. In addition, despite the inclusion of only patients with PSMA-PET uptake, 20–40% of the patients showed little benefit. In the TheraP trial, with more restrictive inclusion with respect to PSMA uptake compared to the VISION trial, ^68^Ga-PSMA-11 and ^18^F-FDG PET/CT scans were performed simultaneously, and 80 of 291 (27%) patients with low PSMA-PET accumulation (SUVmax of lesions less than 20, or SUVmax less than 10 with lesions of more than 10 mm) or FDG-positive were excluded for reasons of having lesions with higher FDG uptake than PSMA uptake. Despite this high selection, 36% of patients did not have an adequate PSA response. Buteau et al. reported a sub-analysis in the TheraP trial and discussed that patients with lower mean 68Ga-PSMA-11 uptake in tumor lesions (PSMA mean) and higher total amount of 18F-FDG uptake in tumor lesions (metabolic tumor volume; MTV) had a lower treatment response [51]. Ferdinandus, J., et al. similarly found that the results of the LuPSMA phase II trial of ^177^Lu-PSMA-617 revealed MTV, mean intensity of PSMA tumor uptake, bone scan index (BSI), ALP, and LDH as biomarkers prognostic of overall survival [52]. Gafita, A., et al. generated a nomogram predicting ^177^Lu-PSMA-617 treatment prognosis using information on 414 ^177^Lu-PSMA-617-treated patients from six studies [53]. The nomogram identified seven factors as prognostic indicators after ^177^Lu-PSMA-617 treatment, including the PSMA mean of the tumor (Table 3). The results of these studies suggest that low tumor PSMA accumulation (PSMA mean) is associated with lower efficacy of Lu-PSMA treatment, but other factors remain to be determined. ^18^F-FDG uptake could be a possible predictor of treatment response, but further studies are needed. Other biochemical (ALP, LDH) and imaging (BSI) parameters that increase with tumor volume correlated with prognosis, but it might simply indicate a selection of patients with a poor prognosis. Thus, although ^177^Lu-PSMA-617 is a promising treatment for CRPC patients, it is difficult to obtain sufficient therapeutic effects even in highly selected patients. Approaches to this problem have included the development of different PSMA ligands, α-ray emitter therapy, and combinations with other agents.

### 3.2. Future Application of PSMA-RLT Agents

#### 3.2.1. PSMA-RLT with β-ray Emitting Radionuclides

Therapeutics with β-ray emitting radionuclides other than ^177^Lu-PSMA-617 are still in the basic or clinical research phase. Clinical trials are underway using different LMW PSMA ligands with PSMA I&T and other short half-life antibodies. In particular, ^177^Lu-PSMA-I&T has been reported to show safety, pharmacokinetics, and tumor uptake comparable to ^177^Lu-PSMA-617 in patients with CRPC, and to prolong the prognosis of HSPC patients with oligometastasis in a small case randomized trial [54]. Open-label randomized controlled phase III trials comparing Lu-PSMA I&T and ARAT in mCRPC patients (NCT04647526) are currently in progress, and results are awaited. ^90^Y-PSMA-617, which employs the β-ray emitting radionuclide ^90^Y, holds the potential to reduce xerostomia and blood toxicity in comparison to 177Lu-PSMA-617 [55]. This suggests that it may be superior in terms of mitigating side effects. We eagerly anticipate future studies to further explore and validate this promising possibility.

#### 3.2.2. PSMA-RLT with α-ray Emitting Radionuclides

Compared to β-rays, α-rays have a shorter range and higher energy, inducing more efficient DSB and causing cancer cell death [45]. Although some case reports have shown dramatic effects [56,57], treatment with α-emitters is still in development regarding safety and availability [58], and we discuss development agents and current issues.

^225^Ac-PSMA-617 has been administered in many treatment-resistant cases of ^177^Lu-PSMA-617 and is the commonly reported agent in PSMA-RLT with α-emitter [59]. In 2016, the first report with Ac-PSMA-617 was published by Kratochwil, C. et al. In this report, two patients with a history of intensive CRPC treatment were treated with 6.4 MBq (100 kBq/kg) of Ac-PSMA-617 for 2–3 cycles, which resulted in a reduction of PSA to less than 0.1 ng/mL [57]. Although there have been some reports of dramatic efficacy, there are few reports evaluating PFS and OS [60]. In addition, it is difficult to evaluate the efficacy of ^225^Ac-PSMA-617 at this time because the number of doses is not stable in each study because of the discontinuation of ^225^Ac-PSMA-617 due to xerostomia and hematologic toxicity [60]. Phase I/II studies with ^225^Ac-PSMA-617 (NCT04597411) and ^225^Ac-PSMA-I&T (NCT04597411, NCT05219500) are currently ongoing, which will clarify the effectiveness, actual frequency of AEs, and dose-limiting toxicity. Furthermore, the supply of ^225^Ac is very small [58] and its dissemination is limited, and ^225^Ac produces toxic daughter nuclides in the decay process [45], which leads to dose limitation due to blood toxicity xerostomia. To overcome the limitations of ^225^Ac, other α-ray RLTs, such as ^161^Tb-PSMA-I&T (NCT05521412) and ^277^Th-PSMA-I&T (NCT03724747) are being evaluated in phase I trials. In terms of productivity and safety, the focus is on ^211^At, ^213^Bi, and ^212^Pb as promising nuclides [45,61].

#### 3.2.3. Combination Therapies

To enhance the therapeutic effect of PSMA-RLT, several trials are underway in combination with various agents. ARATs are one of the most important agents in mHSPC and mCRPC, but at present, the efficacy and safety of the combination with PSMA-RLT agents have not been fully established. Currently, the phase 3 trial of ^177^Lu-PSMA-617 + SoC (including ARAT) in mHSPC (NCT 04720157, PSMAddsion) and the phase 1/2 trial of ^177^Lu-PSMA-617 + enzalutamide in mCRPC (NCT 04419402, ENZA-P) are expected to evaluate the efficacy and safety of the combination therapy. Phase 1/2 trials for combination with taxanes, which are key drugs for CRPC treatment, and ARATs, are also in progress for mCRPC patients (NCT04343885, NCT05340374).

Furthermore, the characteristics of PSMA-RLT as radiotherapy are utilized for combination therapy with other agents. One of the trials was a combination therapy of olaparib, a PARP inhibitor, and ^177^Lu-PSMA-617 (NCT03874884, LuPARP). ^177^Lu causes DNA single-strand breaks by β-rays [45], and PARP contributes to this damage repair [8,62,63]. Therefore, the use of olaparib and 177Lu-PSMA-617 is expected to have a synergistic anti-tumor effect. It has been reported that PSMA expression correlates with DNA damage repair (DDR) mutation [64], and since patients with DDR mutation are more radiosensitive [65] and effective to olaparib [8,62,66,67,68], synergistic effects between both agents could be expected. Clinical trials of combination therapy aimed to exploit the “abscopal” effect, which is a benefit of radiotherapy, are also ongoing. The abscopal effect is considered a therapeutic response to irradiated tumor metastasis. It is caused by the induction of anti-tumor immunity due to exposure to tumor antigens following tumor disruption caused by irradiation of the focal tumor [69]. Therefore, combination therapies of radiation and immune checkpoint inhibitors are being developed to increase the abscopal effect [70,71]. Phase 2 clinical trials for progressive mCRPC patients are currently underway for ^177^Lu-PSMA-617 in combination with pembrolizumab, a PD-1 inhibitor (NCT03658447, PRINCE), and nivolumab and ipilimumab, a PD-1 inhibitor and CTLA4 inhibitor, respectively (NCT05150236, ANZUP2001).

**Figure 1:**
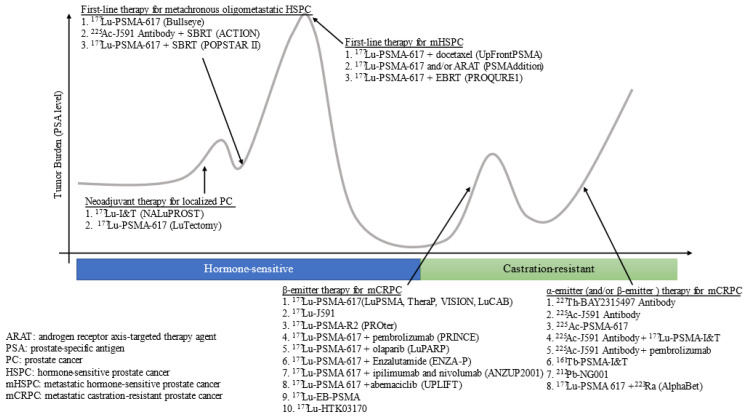
Overview of PSMA theranostics clinical trials as of June 2023.

Finally, RLT combinations of α- and β-emitter agents have been developed. Clinical trials for combination therapy of the α-ray emitting agents ^223^Ra and ^177^Lu-PSMA-I&T (NCT05383079, AlphaBet), ^225^Ac-J591 antibody, and ^177^Lu-PSMA-I&T (NCT04886986) are underway, respectively. As described, the combination of PSMA-RLT with many agents is being investigated. The ongoing clinical trials are listed in Table 4 and Figure 1.

#### 3.2.4. PSMA-Targeted Nanoparticles

Nanoparticles (NPs) such as polymers, DNA polyplexes, lipid particles, liposomes, metals, and proteins have been developed in regard to ligand molecules that target PSMA [72]. NPs targeting PSMA with metals such as iron oxide [73] and gold [74], polymers [75], and liposomes [76] have been developed and are expected to be used clinically in the future.

### 3.3. Clinical Problems in PSMA-RLT

#### 3.3.1. Off-Target Effects

β- and α-emitters have high cytotoxicity and are capable of causing serious damage to normal healthy tissues. The PSMA-RLTs currently in use accumulate mainly in blood, salivary glands, and kidneys [77,78,79]; consequently, disturbances to these tissues need to be considered. Xerostomia is the most typical side effect of PSMA-RLT and has been reported frequently, especially in patients treated with ^225^Ac-PSMA-617 [60,80]. Several attempts have been made to reduce the uptake of radionuclides into the salivary glands, but xerostomia has led to dose limitation in many patients [60]. Hematologic toxicity should also be considered. In the VISION trial, anemia, leukopenia, and thrombocytopenia Grade 3 or 4 were observed in 12.9, 2.5, and 7.9% of patients treated with ^177^Lu-PSMA-617, respectively [10]. An analysis of 106 patients treated with ^225^Ac-PSMA-617 reported Grade 3 or higher anemia, leukopenia, and thrombocytopenia in 0.9, 2.8, and 1.9% of patients, respectively [81]. However, it should be considered that the median cycle is 4 cycles, and the dosage was reduced to 4–6 MBq after the third cycle, while the first two cycles were 8 MBq in this report. It has also been reported that hematologic toxicity appeared more frequently in patients with renal dysfunction. In addition, there is concern about renal dysfunction because ^177^Lu-PSMA-617 and ^225^Ac-PSMA-617 accumulate in the kidneys and are excreted via the urinary tract. The over Grade 3 renal dysfunction frequency was 0.6% and 7% higher for ^225^Ac-PSMA-617 than for ^177^Lu-PSMA-617, respectively [59]. For ^225^Ac-PSMA-617, many reports of a relatively small number of cycles have been reported, and future studies are needed.

#### 3.3.2. Supply of α- and β-Emitters

Until recently, ^177^Lu has been most widely used in the production of ^177^Lu-DOTATATE for the treatment of neuroendocrine tumors. However, after the positive results of the VISION trial, the demand for ^177^Lu has drastically increased. Most of the ^177^Lu produced worldwide is used for both ^177^Lu-PSMA-617 and ^177^Lu-DOTATATE, and a large amount of ^177^Lu is produced in a nuclear reactor through irradiation of source material with neutrons [82]. There is a possibility of a deficit supply of ^177^Lu due to increasing demand, and some efforts are underway to develop a different production method, but it is not yet at a practical stage [83]. Currently, the production of ^225^Ac still relies on extraction from ^229^Th, with only three sources in the world (Directorate for Nuclear Safety and Security of the JRC of the European Commission in Karlsruhe, Germany (formerly known as the Institute for Transuranium Elements) [84], Oak Ridge National Laboratory (ORNL), US [85], and at the Institute of Physics and Power Engineering (IPPE) in Obninsk, Russia) [58], thus, supplies are still limited. New accelerator-based techniques (spallation of ^232^Th, proton and deuteron irradiation of ^226^Ra, and ^226^Ra irradiation based on photonuclear reactions) are being explored to increase ^225^Ac production, but each technique still has its limitations and requires further development [58].

Under current supply circumstances, due to limited production of both ^177^Lu and ^225^Ac, ^177^Lu-PSMA and ^225^Ac-PSMA-RLT will not be able to replace the classical treatment of mCRPC for all patients. Infrastructure development, including strengthening of the supply chain, will be necessary in the future.

#### 3.3.3. Safety and Radiation Protection

^177^Lu-PSMA-617 is an agent with low toxicity and excellent therapeutic efficacy, but previous reports suggest that it is not suitable for some patients. In 2019, the European Association of Nuclear Medicine (EANM) published a guideline for ^177^Lu-PSMA-RLT [86]. The guideline was aimed at patient safety and radiation protection of medical staff based on the Declaration of Helsinki for the unapproved ^177^Lu-PSMA-RLT agents. The guideline identifies six contraindications to the administration of ^177^Lu-PSMA-RLT (Table 5). The majority of clinical trials for ^177^Lu-PSMA-RLT are designed according to this guideline, and the efficacy and safety of PSMA-RLT have not been established in patients not meeting these criteria. When actually administering ^177^Lu-PSMA-RLT to the patient, these contraindications need to be confirmed. Especially in patients with uncontrolled urinary tract obstruction, hydronephrosis, or at risk for urinary retention, delayed excretion of ^177^Lu-PSMA-RLT possibly leads to nephrotoxicity and other side effects. In addition, drug interactions between ^177^Lu-PSMA-RLT and other CRPC drugs have not been validated, and more than four weeks is recommended between the last dose of chemotherapy, ARAT, or bone tracer and administration of ^177^Lu-PSMA-RLT.

Patients with urinary incontinence are more difficult to manage from the viewpoint of radiation protection. In these patients, because most ^177^Lu-PSMA-RLT agents are excreted in the urine, there is the possibility of increased risk of exposure to health care providers and caregivers compared to other patients. Strategies to prevent this problem need to be fully considered prior to introducing treatment.

#### 3.3.4. Patients with Better Indications for PSMA-RLT Agents

PSMA-RLT is difficult to adapt to all imaging-selected CRPC patients due to supply limitations at present. Therefore, it is preferred to be able to highly select patients who are more likely to benefit from PSMA-RLT. High PSMA-PET and low FDG-PET accumulation are considered to be promising selection criteria. It is impossible to determine from current reports at which stage of prostate cancer PSMA-RLT should be administered.

The use of these agents should also be considered from a safety perspective. Because of urinary excretion, ^177^Lu-PSMA-617 is not safe to administrate in patients with uncontrolled urinary tract obstruction, hydronephrosis, or at risk for urinary retention due to delayed excretion. There are also still unclear drug interactions, and it is necessary to carefully check concomitant medications. It should be considered that the metabolization of PSMA-RLT has the potential to change depending on the type of nuclide and ligand, and that a wide variety of agents are expected to be developed in the future, and their administration should take into account the patient’s condition. Finally, in terms of radiation protection, we believe that the possibility of increased exposure risk to medical personnel and caregivers should be fully considered in patients who present with severe urinary incontinence.

## 4. Conclusions and Future Directions

Recently, PSMA-RLT has dramatically altered the treatment of mCRPC and is a feasible alternative to conventional treatment. As a result, the development of agents targeting PSMA is expected to accelerate in radiopharmaceutical research. The approval of ^68^Ga-PSMA-11 and ^177^Lu-PSMA-617 as a therapeutic by the FDA and EMA should accelerate this trend. Several clinical trials are still underway to develop treatments with different PSMA ligands, such as ^177^Lu-PSMA-I&T and short-lived antibodies, as well as α-emitters. However, there are problems that must be overcome in the current PSMA-RLT. In particular, issues of supply, side effects, and the type of patients to whom PSMA-RLT should be applied are critical to its future development. It will be necessary to address these problems through the development of various agents and clinical trials in the future.

## Figures and Tables

**Table 1:** (**A**) Qualitative evaluation of PSMA expression on a four-point scale. (**B**) miTNM classification.

(A)	
**Regional Classification of PSMA-PET Findings**	
Class Description	
Local tumor (T)	
miT0	No local tumor
miT2	Organ-confined tumor
miT3a	Non-organ-confined tumor (extracapsular extension)
miT3b	Non-organ-confined tumor (seminal vesicles invasion)
miT4	Tumor invading adjacent structures (other than seminal vesicles)
miTr	Presence of local recurrence after radical prostatectomy
Regional nodes (N)	
miN0	No positive regional lymph nodes
miN1	Positive regional lymph nodes
Distant metastases (M)	
miM0	No distant metastases
miM1a	Extra-pelvic lymph nodes
miM1b	Bone metastasis
miM1c	Non-nodal visceral metastasis: report involved organ(s)
(**B**)	
**Regional Classification of PSMA-PET Findings**	
Class Description	
Local tumor (T)	
miT0	No local tumor
miT2	Organ-confined tumor
miT3a	Non-organ-confined tumor (extracapsular extension)
miT3b	Non-organ-confined tumor (seminal vesicles invasion)
miT4	Tumor invading adjacent structures (other than seminal vesicles)
miTr	Presence of local recurrence after radical prostatectomy
Regional nodes (N)	
miN0	No positive regional lymph nodes
miN1	Positive regional lymph nodes
Distant metastases (M)	
miM0	No distant metastases
miM1a	Extra-pelvic lymph nodes
miM1b	Bone metastasis
miM1c	Non-nodal visceral metastasis: report involved organ(s)

Adapted from [40].

**Table 2:** List of selected clinically available α- and β-emitters and their characteristics.

Alpha Emitters: Physical Properties			
Radionuclide	E_average_ (MeV)	Range (µm)	Half-life
211At	6.79	60	7.2 h
213Bi	8.32	84	46 min
223Ra	5.64	45	11.43 d
225Ac	6.83	61	10 d
Beta emitters: physical properties			
Radionuclide	Energy_max_ (keV)	Range (mm)	Half-life
177Lu	497	1.8	6.7 d
67Cu	575	2.1	61.9 h
131I	606	2.3	8.0 d
90Y	2284	11.3	64.1 h

**Table 3:** Prognostic factors after treatment with ^177^Lu-PSMA-617.

1	Time since diagnosis (years)
2	Chemotherapy status previous chemotherapy (yes/no)
3	Haemoglobin (g/dL)
4	Tumor SUVmean of PSMA-PET
5	Number of lesions (<20/≥20)
6	Bone metastases (yes/no)
7	Liver metastases (yes/no)

Adapted from [53]. SUV = standardized uptake value.

**Table 4:** Summary Clinical Trials of PSMA-RLT.

Clinical Trial Identifier	Brief Description of the Trials	Phase
	Trials for Localized Prostate Cancer	
NCT04297410	^177^Lu-PSMA-I&T prior to prostatectomy (NALuPROST)	1/2
NCT04430192	^177^Lu-PSMA-617 prior to prostatectomy (LuTectomy)	1/2
	Trials for mHSPC	
NCT04343885	^177^Lu-PSMA-617 + docetaxel vs. docetaxel in mHSPC (UpFrontPSMA)	2
NCT04443062	^177^Lu-PSMA-617 in oligometastatic metachronous HSPC (Bullseye)	2
NCT04506567	^225^Ac-J591 antibody + SBRT or ^225^Ac-J591 Antibody + ADT in oligometastatic metachronous HSPC (ACTION)	1/2
NCT04720157	^177^Lu-PSMA-617 + SOC vs. SOC alone in mHSPC (PSMAddition)	3
NCT05079698	^177^Lu-PSMA-617 + SBRT in oligometastatic metachronous HSPC	1
NCT05162573	^177^Lu-PSMA-617 + EBRT in N1M0 mHSPC (PROQURE-1)	1
NCT05560659	^177^Lu-PSMA-617 + SBRT vs. SBRT in oligometastatic metachronous HSPC (POPSTAR II)	2
	Trials for mCRPC	
ACTRN12615000912583	^177^Lu-PSMA-617 in progressive mCRPC (LuPSMA)	2
NCT00538668	^177^Lu-J591 antibody in progressive mCRPC	1
NCT03392428	^177^Lu-PSMA-617 vs. cabazitaxel in progressive mCRPC (TheraP)	2
NCT03490838	^177^Lu-PSMA-R2 in progressive mCRPC(PROter)	1/2
NCT03511664	^177^Lu-PSMA-617 + SOC vs. SOC in progressive mCRPC (VISION)	3
NCT03658447	^177^Lu-PSMA-617 + pembrolizumab in progressive mCRPC (PRINCE)	1/2
NCT03724747	^227^Th-BAY2315497 antibody in progressive mCRPC	1
NCT03874884	^177^Lu-PSMA-617 + olaparib in progressive mCRPC (LuPARP)	1
NCT04419402	Enzalutamide + ^177^Lu-PSMA-617 vs. enzalutamide alone in mCRPC (ENZA-P)	2
NCT04506567	^225^Ac-J591 antibody in progressive mCRPC	1/2
NCT04597411	^225^Ac-PSMA-617 in progressive mCRPC	1
NCT04647526	^177^Lu-PSMA-I&T vs. ARAT in progressive mCRPC (SPLASH)	3
NCT04663997	^177^Lu-PSMA-617 vs. docetaxel in progressive mCRPC	2
NCT04886986	^225^Ac-J591 antibody + ^177^Lu-PSMA-I&T in progressive mCRPC	1/2
NCT04946370	^225^Ac-J591 antibody + pembrolizumab in progressive mCRPC	1/2
NCT04996602	^177^Lu-EB-PSMA in progressive mCRPC	1
NCT05150236	^177^Lu-PSMA-617 + ipilimumab and nivolumab vs. ^177^Lu-PSMA-617 in progressive mCRPC (ANZUP2001)	2
NCT05113537	^177^Lu-PSMA-617 + abemaciclib in progressive mCRPC (UPLIFT)	1/2
NCT05521412	^161^Tb-PSMA-I&T in progressive mCRPC (VIOLET)	1/2
NCT05219500	^225^Ac-PSMA-I&T in progressive mCRPC (TATCIST)	2
NCT05340374	^177^Lu-PSMA-617 + cabazitaxel in mCRPC (LuCAB)	1/2
NCT05383079	^177^Lu-PSMA-I&T + radium-223 in progressive mCRPC (AlphaBet)	1/2
NCT05570994	^177^Lu-HTK03170 in progressive mCRPC	1/2
NCT05725070	^212^Pb-NG001 in progressive mCRPC	1

**Table 5:** Contraindications of ^177^Lu-PSMA-RLT in the EANM procedure guideline.

1	Life expectancy is less than 6 months (ECOG performance status > 2); unless the main objective is alleviating suffering from disease-related symptoms.
2	Unacceptable medical or radiation safety risk for isolation on a nuclear medicine therapy unit (if required by national regulations).
3	Unmanageable urinary tract obstruction or hydronephrosis; in patients with diagnosed or who are at high risk of urinary retention, 99mTc-MAG3 or 99mTc-DTPA renal scintigraphy should be considered as a baseline exam.
4	Progressive deterioration of organ function (GFR < 30 mL/min or creatinine > 2-fold upper limit of normal (ULN); liver enzymes > 5-fold ULN).
5	Myelosuppression:
	a. Total white cell count less than 2.5 × 10^9^/L
	b. Platelet count less than 75 × 10^9^/L
6	Conditions that require timely interventions (radiation therapy, surgery), e.g., spinal cord compression and unstable fractures, PSMA-RLT might be performed afterward upon the patient’s condition. Borderline cases should be evaluated within the multidisciplinary tumor board for the individual benefit-to-risk ratio.

Adapted from [46].
